# Association between mobile phone use and inattention in 7102 Chinese adolescents: a population-based cross-sectional study

**DOI:** 10.1186/1471-2458-14-1022

**Published:** 2014-10-01

**Authors:** Feizhou Zheng, Peng Gao, Mindi He, Min Li, Changxi Wang, Qichang Zeng, Zhou Zhou, Zhengping Yu, Lei Zhang

**Affiliations:** Department of Occupational Health, Key Laboratory of Medical Protection for Electromagnetic Radiation, Ministry of Education, Third Military Medical University, NO. 30 Gaotanyan Street, Chongqing, 400038 People’s Republic of China; Suining Experimental High School, Sichuan, 629000 People’s Republic of China; Dongchan Middle School, Sichuan, 629007 People’s Republic of China

**Keywords:** Mobile phone, Inattention, Chinese adolescents, Cross-sectional study

## Abstract

**Background:**

The dramatic growth of mobile phone (MP) use among young people has increased interest in its possible health hazards in this age group. The aim of this cross-sectional study was to investigate the association between MP use and inattention in adolescents.

**Methods:**

A total of 7720 middle school students were involved in this cross-sectional study. Inattention was assessed as defined for the Attention Deficit component of Attention deficit/Hyperactivity disorder (ADHD) by the Diagnostic and Statistical Manual of Mental Disorders (4th ed., text rev. [DSM-IV-TR]). The demographic characteristics and information on MP use were included in the questionnaire. Chi-square tests and logistic regression models were used to analyze the data.

**Results:**

In total, 7102 (91.99%) valid questionnaires were obtained. After adjusted for confounders, inattention in adolescents was significantly associated with MP ownership, the time spent on entertainment on MP per day, the position of the MP during the day and the mode of the MP at night. The strongest association between inattention and the time spent on the MP was among students who spent more than 60 minutes per day playing on their MP.

**Conclusions:**

Our study shows some associations between MP use and inattention in Chinese adolescents. Decreasing MP usage to less than 60 minutes per day may help adolescents to stay focused and centered.

## Background

With the incorporation of modern electronic products into daily life, adolescents now have a longer lifetime exposure to mobile phone (MP). China has developed into one of the world's largest MP markets. As today’s adolescents frequently use MP and other communication tools in their homes, community environments and at school [1], they have longer exposure time to MP. In 2014, the proportion of adolescents who own MP is more than 60% in Shanghai, China [2] and still increasing. Additionally, with the increasing use of MP, concerns have been raised in a number of countries about the adverse health effects of MP use on adolescents. World Health Organization (WHO) has identified studies on the potential health effects of MP in children and adolescents as a high priority research area in their research agenda for radiofrequency fields [3].

Making calls, sending messages, surfing the internet and playing games on MP are very common in adolescents’ lives, as is the wide use of smart phones. By the end of 2013, there were a total of 500 million people using MP to browse the internet in China [4]. It is unclear if MP use has adverse physiological and psychological effects on the development of adolescents. Inattention is one of the most prevalent mental health disorders in adolescents [5]. Inattentive symptoms, in particular, were strongly associated with problematic video game use or overuse also with other media [6]. These popularly operated MP games, often in brief segments, are not attention demanding and offer immediate rewards, which may encourage further playing [7]. Exposure to MP radio frequency electromagnetic fields might affect nonspecific neurologic performance such as attention and cognition [8]. Additionally, children absorb more energy from external electromagnetic fields than adults [9]. A growing number of studies have focused on the harmful effects of exposure to MP; however, only a few have investigated the association between inattention in adolescents and MP use.

MP has been found to be associated with inattention in a few studies [10, 11]. One explanation of this association could be that the head is more exposed to electromagnetic radiation from MP rather than any other part of the body. Alternatively, subjects suffering from insomnia [12] or headaches [13] were found to have more inattention and many studies have reported that insomnia [14, 15] or headaches [16, 17] occur more frequently with increasing exposure to MP. Furthermore, adolescents with inattention are at a higher risk of other psychiatric illnesses such as mood and conduct disorders, and substance abuse [18, 19]. However, in a study using a MP exposure device, no difference in attention was observed between the sham and MP exposure groups [20, 21]. Attention functions may also be differentially enhanced after exposure to the electromagnetic field emitted by MP [22–24].

Although there have been several studies on the association between MP use and attention, the results were still controversial. Our present study investigated the possible association between MP use and inattention in Chinese adolescents using a cross-sectional design.

## Methods

### Ethics statement

The protocol of this study was approved by the Third Military Medical University Ethical Committee. All study participants obtained written consent from their parents or guardians.

### Subjects

In this cross-sectional survey, questionnaires were sent out to 7720 currently enrolled students from 4 middle schools in southwestern China. After obtaining written consent from the students' parents or guardians, the questionnaires were distributed and collected during school hours by the research staff who had previously received epidemiological survey training. The students could ask the research staff if they had any problems with the questions while they filled out the survey in the presence of their class teacher. Among the 7426 (96.19%) students who responded to the questionnaire, 7102 (91.99%) valid questionnaires were analyzed after excluding those with incomplete information.

### Questionnaire

The questionnaire used in our research was designed to capture information about demographics, MP use, and inattention.

### Demographic information

In the section on demographic information, name, sex (male or female), age, school, grade (7–12), and address (urban/rural) were listed.

### Information on MP use

To obtain information about the time spent using a MP, students were asked to answer the following questions: “Do you own a MP?”, “At what age did you start using a MP?”, “How much time do you spend making phone calls per day?”, and “How much time do you spend on MP entertainment (playing games and browsing the internet) per day?”. MP usage was assessed using these questions: “How do you answer the phone (hold it close to your ear, hands-free, or use headphones)?”; “Where do you put your MP during the day (not carrying, hanging in front of the chest, in coat pockets, in trouser pockets, or in bags)?”; and “What is the mode of your MP at night (powered on and beside your head, powered on and kept away from your head, or powered off)?”. For all the above questions, MP use included using other people’s phones. The question “Is there a mobile base station around your home or school?” was asked as well. Additionally, their answers would be checked with the information about the address of mobile base station provided by the Telco Providers and the consistent answers were used for analysis.

### Inattention

Inattention was described as a lack of attention or a reduced attention span. Some examples of inattention include: avoiding school projects (which involve a long periods of concentration); losing school supplies; difficulties completing household chores; easily distractible et al. The prevalence of inattention in our study was screened using the most stable psychometric properties of the Attention Deficit component of Attention deficit/Hyperactivity disorder (ADHD) by the Diagnostic and Statistical Manual of Mental Disorders (4th ed., text rev. [DSM-IV-TR]) [25–27]. “A” criteria were used by the teachers who had previously received epidemiological survey training. The choice of nine inattention descriptions was “yes/no”. Inattention was defined when the teacher chose six or more “yes” responses to the descriptions.

### Statistical analysis

Chi-squared tests (*χ*^2^) were used to compare the prevalence of inattention between different classifications of MP usage. Odds ratios (OR) and 95% confidence intervals (95% CI) were obtained using logistic regression models to assess the possible associations between MP ownership, years of MP usage, minutes spent on calls each day, minutes spent on entertainment each day, habit of answering the phone, position of MP during the day, mode of MP at night and the prevalence of inattention in adolescents. Adjusted OR were also calculated after adjusting for age, sex, urban/rural residence and whether living close to mobile base stations. The variables with P < 0.1 were included in logistic regression models used to assess the association. Assessment of the fit between the model and the data was gauged by the goodness-of-fit test and the log likelihood Chi-square test. Continuous variables, such as the years of MP usage, the minutes spent on calls daily and the time of entertainment, were split into tertiles. Choosing six or more “yes” of the inattention descriptions was defined inattention. Statistical significance was defined as P < 0.05 in this study. Categorical variables were summarized using the corresponding percentages, and continuous variables were generally summarized using descriptive statistics (mean ± standard deviation (SD)). Statistical analysis was undertaken using SPSS version 19.0 (SPSS Inc., Chicago, IL, USA).

## Results

### Descriptive information

Out of 7720 currently enrolled students in the 4 middle schools, 294 (3.81%) did not return the questionnaire. Out of the 7426 returned questionnaires, 324 (4.20%) were incomplete. In total, 7102 (91.99%) questionnaires completed in all sections (including the Demographic information, Information on MP, and Inattention) were used in the analysis. The participants included 3613 males (50.87%) and 3489 females (49.13%). The mean age was 15.26 ± 1.77 years. A total of 5033 (70.87%) of the participants resided in urban areas, and 2069 (29.13%) were from rural areas.

Overall, 5668 (79.81%) participants owned MP at the time of the survey and had been using a MP for a mean of 3.50 ± 2.48 years. Participants spent 57.36 ± 71.96 minutes on entertainment and 8.64 ± 15.48 minutes on making calls daily (Table 1). The details of the socio-demographic characteristics and MP usage are given in Table 1.

**Table 1:** **Descriptive data of socio**-**demographic characteristics and MP usage** (**N** = **7102**)

Characteristic	Prevalence n (%)	
**Sex**		
Male	3613 (50.87)	
Female	3489 (49.13)	
**Age**		
12	248 (3.49)	
13	968 (13.63)	
14	1340 (18.87)	
15	1355 (19.08)	
16	1119 (15.76)	
17	906 (12.76)	
18	669 (9.42)	
19	194 (2.73)	
20	19 (0.27)	
**Urban**/**Rural**		
Rural	2069 (29.13)	
Urban	5033 (70.87)	
**MP Ownership**		
Own MP	5668 (79.81)	
Don't own MP	1434 (20.19)	
**Whether close to mobile base stations**		
Close to mobile base stations	3920 (55.20)	
Far away from mobile base stations	3182 (44.80)	
**Characteristic**	**Mean** ± **SD**	**Min**-**Max**
Age	15.26 ± 1.77	12-20
MP use years	3.50 ± 2.48	0-18
Minutes on call (min/day)	8.64 ± 15.48	0-180
Time of entertainment (min/day)	57.36 ± 71.96	0-480

### Association between MP use and inattention

There were 7294 (94.48%) responses to the inattention questions. The overall prevalence of inattention was 69.79% out of the 7102 valid questionnaires in this study. After adjusted for age, sex, area of residence (urban/rural) and whether living close to mobile base stations, the prevalence of inattention was significantly associated with MP ownership (OR 2.92; 95% CI 2.51-3.39) and time spent on entertainment daily (OR 1.87; 95% CI 1.28-2.73). Additionally, there was a positive association between inattention and the time spent on entertainment on MP (21–60 minutes per day spent on entertainment, OR 1.45, 95% CI 1.06-1.97; >60 minutes per day spent on entertainment, OR 1.82, 95% CI 1.28-2.59; Table 2). We analyzed the association between inattention and the position of MP during the day. The results showed significant differences. Compared to not carrying the MP (OR 1.00), hanging the MP in front of the chest (OR 0.44; 95% CI 0.19-0.99) and putting the MP in a trouser pocket (OR 1.34; 95% CI 1.10-1.62) were both significantly associated with inattention. Moreover, participants who powered off their MP at night showed significantly less inattention than those students who left their MP on at night (OR 0.75; 95% CI 0.63-0.90; Table 3).

**Table 2:** **Association between MP use time and inattention** (**n** = **7102**)

	n (%)	P ^^^	OR (95% CI)	Adjusted OR (95% CI)^#^
**MP ownership**		0.000		
No (ref)	541 (38.21)		1.00	1.00
Yes	4414 (79.29)		5.73 (5.05-6.50)	2.92 (2.51-3.39)**
**MP use years**		0.095		
0-2 years (ref)	1568 (76.34)		1.00	1.00
3-4 years	1444 (79.04)		1.16 (0.99-1.35)	1.07 (0.88-1.31)
> 4 years	1132 (78.61)		1.14 (0.97-1.34)	1.05 (0.85-1.30)
**Minutes on call**		0.000		
0-2 years				
0-1 min/day (ref)	344 (81.32)		1.00	1.00
1-6 min/day	594 (79.41)		0.87 (0.64-1.18)	1.15 (0.82-1.60)
> 6 min/day	410 (76.64)		0.76 (0.55-1.06)	1.16 (0.81-1.65)
3-4 years				
0-1 min/day	327 (82.78)		1.05 (0.73-1.51)	1.06 (0.72-1.57)
1-6 min/day	495 (82.14)		1.07 (0.77-1.49)	1.18 (0.83-1.68)
> 6 min/day	398 (80.08)		0.95 (0.68-1.34)	1.31 (0.90-1.91)
>4 years				
0-1 min/day	261 (79.33)		0.86 (0.60-1.25)	0.92 (0.62-1.37)
1-6 min/day	378 (83.44)		1.23 (0.86-1.76)	1.46 (0.99-2.17)
> 6 min/day	338 (78.42)		0.81 (0.58-1.14)	1.12 (0.77-1.64)
**Time of entertainment**		0.000		
0-2 years				
0-20 min/day (ref)	548 (79.42)		1.00	1.00
21-60 min/day	459 (79.69)		1.03 (0.78-1.36)	1.45 (1.06-1.97)*
> 60 min/day	317 (78.66)		0.98 (0.72-1.32)	1.82 (1.28-2.59)**
3-4 years				
0-20 min/day	410 (82.66)		1.22 (0.90-1.64)	1.11 (0.80-1. 53)
21-60 min/day	440 (82.09)		1.18 (0.88-1.58)	1.33 (0.97-1.83)
> 60 min/day	353 (80.78)		1.19 (0.87-1.62)	1.80 (1.27-2.56)**
>4 years				
0-20 min/day	334 (76.78)		0.98 (0.72-1.32)	0.99 (0.71-1.38)
21-60 min/day	325 (82.70)		1.28 (0.92-1.78)	1.39 (0.97-1.98)
> 60 min/day	294 (80.55)		1.11 (0.80-1.53)	1.87 (1.28-2.73)*

P^^^obtained through *χ* ^2^ test.

^#^Adjusted for sex, age, Urban/Rural residence and whether living close to mobile base stations.

*P < 0.05. **P < 0.001.

OR: odds ratio, 95% CI: 95% confidence interval.

**Table 3:** **Association between MP use status and inattention** (**n** = **7102**)

	n (%)	P ^^^	OR (95% CI)	Adjusted OR (95% CI)^#^
**Habit of answering the phone**		0.798		
Close to ear (ref)	2616 (80.20)		1.00	1.00
Hands-free	783 (80.72)		0.99 (0.82-1.18)	1.07 (0.88-1.29)
Use headphone	567 (81.23)		1.02 (0.83-1.26)	1.04 (0.83-1.30)
**Position of MP during the day**		0.000		
Do not carry (ref)	401 (84.24)		1.00	1.00
Hang in front of chest	12 (44.44)		0.24 (0.12-0.46)**	0.44 (0.19-0.99)*
In coat pockets	239 (77.60)		1.14 (0.87-1.49)	1.28 (0.95-1.73)
In trouser pockets	1080 (78.89)		0.98 (0.83-1.15)	1.34 (1.10-1.62)*
In bags	348 (85.50)		1.14 (0.88-1.47)	1.04 (0.78-1.37)
**Mode of MP at night**		0.000		
Power on and beside their heads (ref)	922 (52.12)		1.00	1.00
Power on and keep away from their heads	466 (42.02)		0.93 (0.77-1.13)	0.87 (0.71-1.06)
Power off	612 (32.74)		0.86 (0.72-1.01)	0.75 (0.63-0.90)*

P^^^obtained through *χ* ^2^ test.

^#^Adjusted for sex, age, Urban/Rural residence and whether living close to mobile base stations.

*P < 0.05. **P < 0.001.

OR: odds ratio, 95% CI: 95% confidence interval.

## Discussion

This population-based cross-sectional study is one of the first studies to investigate the association between MP use and inattention in adolescents in China. Our results showed that the prevalence of inattention was considerable among middle school students. In this study, inattention in adolescents was significantly associated with MP ownership, the time spent on entertainment on the MP every day, the position of the MP during the day and the mode of the MP at night.

The prevalence of inattention in the present study was much higher than previous attention studies whether related to MP use or not [28, 29]. Compared with the two previous studies investigating several symptoms including headache, fatigue and dizziness, our study focused only on inattention. This might have led the students to focus on this one symptom, resulting in the high prevalence of reported inattention. In contrast, the Mortazavi's study indicated that people in Iran are usually less familiar with the health effects of exposure to electromagnetic fields, therefore, the number of individuals reporting subjective symptoms was considerably lower [29]. The higher prevalence of inattention in our study compared to other studies was also likely due to the higher prevalence of MP ownership (79.82%) in our study than the Iran study (30%).

Our results showed that the prevalence of inattention was significantly higher in MP-owning students compared to non-MP students. This finding concurs with Mortazavi's other studies which showed that there was a statistically significant relationship between the use of cell phones and attention disorder [30, 31]. However, in their previous research, they did not find a significant association between MP use and self-reported symptoms [29]. We also demonstrated that the OR of inattention increased with the duration of time spent on entertainment on the MP per day. A longitudinal study also reported a similar time-dependent association between playing games on a mobile phone and attention disorder [10]. Attention deficit was associated with a weaker function and structure of prefrontal cortex circuits [32]. Moreover, Aalto et al. revealed an increase in regional cerebral blood flow more distantly in the prefrontal cortex while a mobile phone was in operation placed beside the subject's head [33]; this could be a reason for the increase in inattention. Because the time spent on making calls per day was not significantly associated with inattention, the effect of the MP on attentiveness might not be directly from the MP electromagnetic exposure but from the psychological impact. Inattention was found to be related to depression, anxiety, stress [34] and youth violence [35] in previous studies. A relationship between playing computer games [27, 36], internet addiction [37, 38] and inattention has also been described. Moreover, superficial way to use internet or the contents of the games could also cause problems with concentrating. The time spent on games might also exacerbate ADHD symptoms, if not directly then through the loss of time spent on more developmentally challenging tasks [7]. In our present study, the strongest association between inattention and time spent on entertainment on the MP was among participants who spent more than 60 minutes per day playing on their MP. This is the first study so far to determine a time period which is correlated with inattention. Therefore, our results may provide a reference for further research into the relationship between MP use and inattention.

Hanging a MP in front of the chest and putting a MP into trouser pockets were both significantly associated with inattention. However, as only a small group of students (4.34%) hang their MP in front of their chest, the association with inattention may not be generalizable. Our study also showed that putting MP into trouser pockets was likely to increase inattention in MP users. The side-pocket was the favored location for cell phones and students often send texts from inside a pocket. As the exposure increases rapidly in the near field, the safety limits may be exceeded when the phone makes contact with the base station and the penetration of the energy may increase with proximity if MP is in a pocket next to the carrier’s body [39]. The poorer attention in those carrying the MP in a pocket might be due to students using MP while it was in the pocket. As our study found that adolescents who kept their MP turned off at night had significantly less inattention, we propose that parents should power off adolescents' MP while they sleep.

This large-scale cross-sectional study is the first to investigate the association between exposure to MP and inattention in Chinese adolescents. Because the questionnaire survey was proceeded during class time, there was a high response rate among the adolescents. Detailed MP usage and inattention were collected to fully explore the association. Furthermore, to exclude confounding factors, we adjusted the results for sex, age, urban/rural residence and whether living close to mobile base stations.

However, in this study, there may have been some exposure misclassification [40] due to the data being self-reported. Another limitation was that the cross-sectional study design could not adequately reveal the causality of the factors [41]. On the other hand, as the protection of privacy for parents, schools only allowed us to collected the basic information of adolescents, such as sex, age, address, etc. Therefore, the confounding factors in this study were insufficient.

## Conclusions

In general, the results in the present study indicated that MP ownership, the time spent on entertainment on the MP, the position of the MP during the day and the mode of the MP at night were all significantly associated with inattention in Chinese adolescents. We suggest that parents should set a maximum of 60 minutes daily playing by adolescents’ on a MP and require them to turn it off when they sleep.

## Abbreviations

MP: Mobile phone
WHO: World Health Organization
ADHD: Attention deficit/Hyperactivity disorder
OR: Odds ratio
CI: Confidence interval
SD: Standard deviation.
